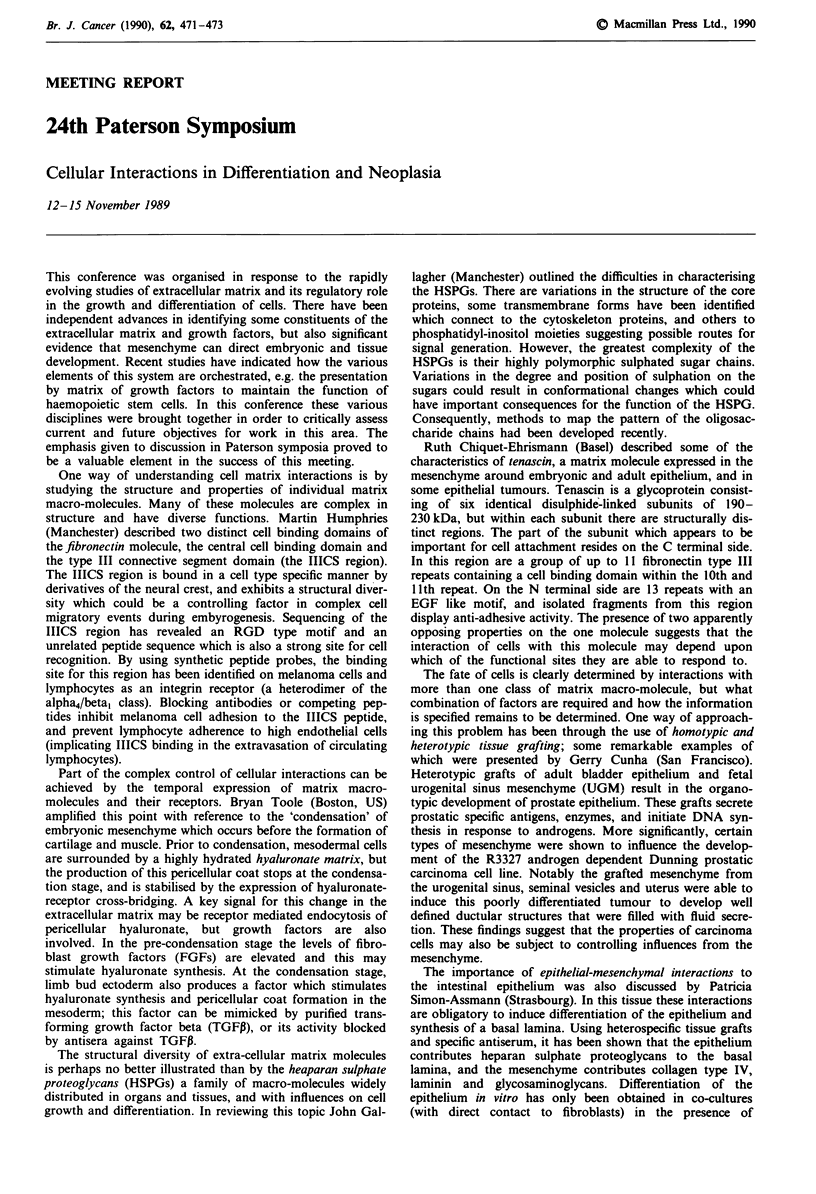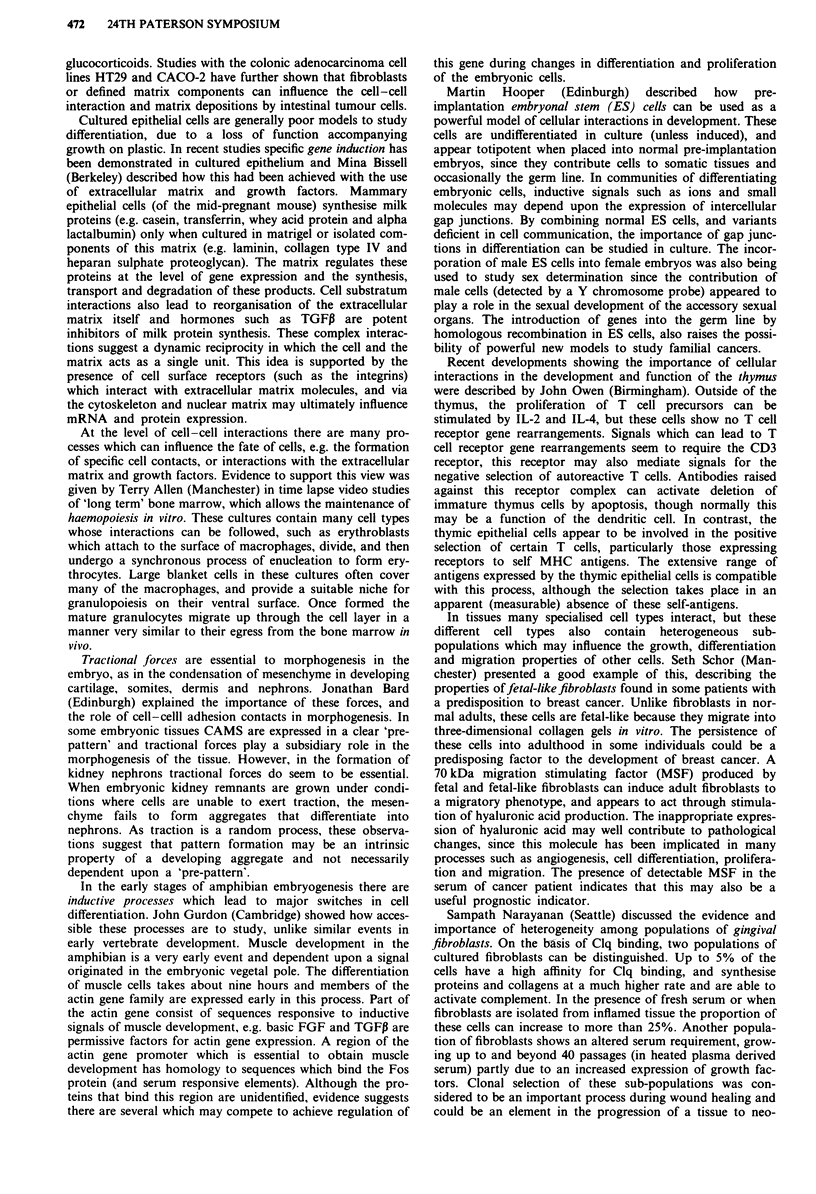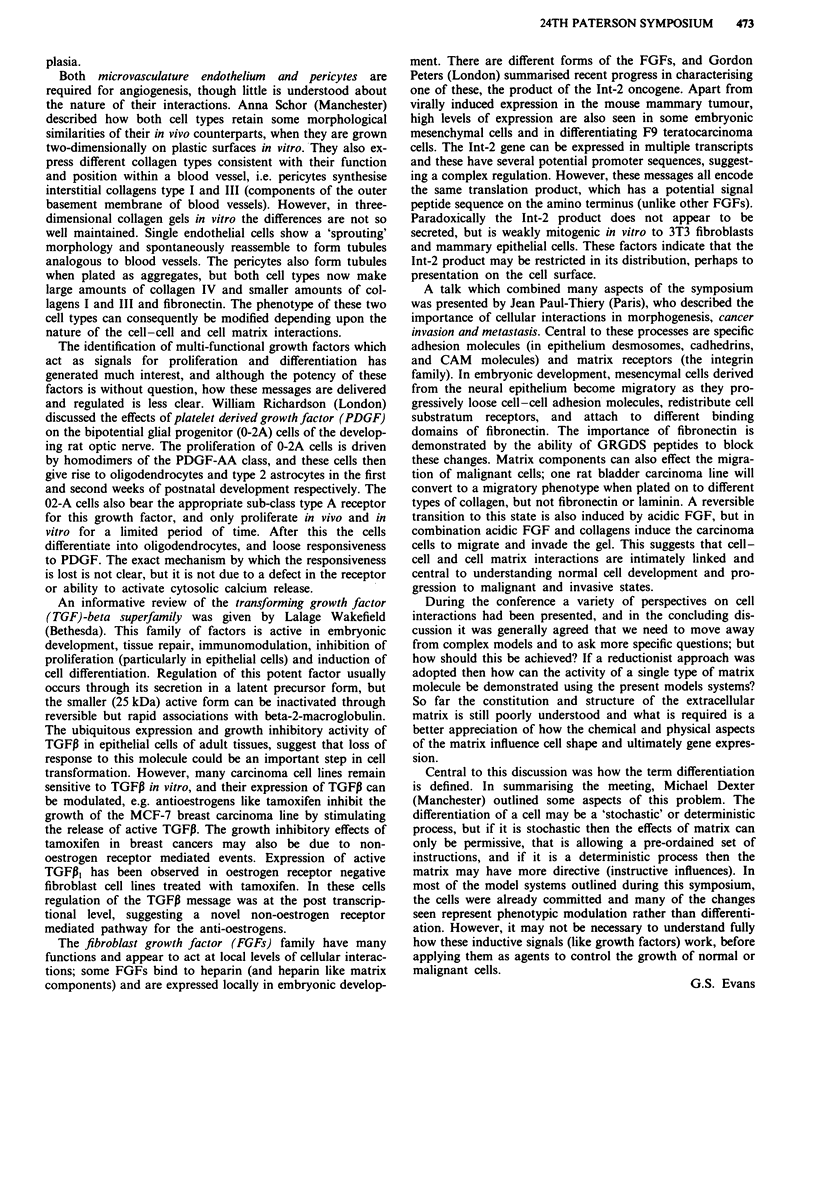# 24th Paterson Symposium. Cellular interactions in differentiation and neoplasia. 12-15 November 1989.

**DOI:** 10.1038/bjc.1990.323

**Published:** 1990-09

**Authors:** G. S. Evans


Br. J. Cancer (1990), 62, 471–473 © Macmillan Press Ltd., 1990

MEETING REPORT

### 24th Paterson Symposium

### Cellular Interactions in Differentiation and Neoplasia

*12–15 November 1989*

This conference was organised in response to the rapidly evolving studies of extracellular matrix and its regulatory role in the growth and differentiation of cells. There have been independent advances in identifying some constituents of the extracellular matrix and growth factors, but also significant evidence that mesenchyme can direct embryonic and tissue development. Recent studies have indicated how the various elements of this system are orchestrated, e.g. the presentation by matrix of growth factors to maintain the function of haemopoietic stem cells. In this conference these various disciplines were brought together in order to critically assess current and future objectives for work in this area. The emphasis given to discussion in Paterson symposia proved to be a valuable element in the success of this meeting.

One way of understanding cell matrix interactions is by studying the structure and properties of individual matrix macro-molecules. Many of these molecules are complex in structure and have diverse functions. Martin Humphries (Manchester) described two distinct cell binding domains of the *fibronectin* molecule, the central cell binding domain and the type III connective segment domain (the IIICS region). The IIICS region is bound in a cell type specific manner by derivatives of the neural crest, and exhibits a structural diversity which could be a controlling factor in complex cell migratory events during embyrogenesis. Sequencing of the IIICS region has revealed an RGD type motif and an unrelated peptide sequence which is also a strong site for cell recognition. By using synthetic peptide probes, the binding site for this region has been identified on melanoma cells and lymphocytes as an integrin receptor (a heterodimer of the $alpha_4/beta_1$ class). Blocking antibodies or competing peptides inhibit melanoma cell adhesion to the IIICS peptide, and prevent lymphocyte adherence to high endothelial cells (implicating IIICS binding in the extravasation of circulating lymphocytes).

Part of the complex control of cellular interactions can be achieved by the temporal expression of matrix macromolecules and their receptors. Bryan Toole (Boston, US) amplified this point with reference to the 'condensation' of embryonic mesenchyme which occurs before the formation of cartilage and muscle. Prior to condensation, mesodermal cells are surrounded by a highly hydrated *hyaluronate matrix*, but the production of this pericellular coat stops at the condensation stage, and is stabilised by the expression of hyaluronate-receptor cross-bridging. A key signal for this change in the extracellular matrix may be receptor mediated endocytosis of pericellular hyaluronate, but growth factors are also involved. In the pre-condensation stage the levels of fibroblast growth factors (FGFs) are elevated and this may stimulate hyaluronate synthesis. At the condensation stage, limb bud ectoderm also produces a factor which stimulates hyaluronate synthesis and pericellular coat formation in the mesoderm; this factor can be mimicked by purified transforming growth factor beta (TGF$\beta$), or its activity blocked by antisera against TGF$\beta$.

The structural diversity of extra-cellular matrix molecules is perhaps no better illustrated than by the *heaparan sulphate proteoglycans* (HSPGs) a family of macro-molecules widely distributed in organs and tissues, and with influences on cell growth and differentiation. In reviewing this topic John Gallagher (Manchester) outlined the difficulties in characterising the HSPGs. There are variations in the structure of the core proteins, some transmembrane forms have been identified which connect to the cytoskeleton proteins, and others to phosphatidyl-inositol moieties suggesting possible routes for signal generation. However, the greatest complexity of the HSPGs is their highly polymorphic sulphated sugar chains. Variations in the degree and position of sulphation on the sugars could result in conformational changes which could have important consequences for the function of the HSPG. Consequently, methods to map the pattern of the oligosaccharide chains had been developed recently.

Ruth Chiquet-Ehrismann (Basel) described some of the characteristics of *tenascin*, a matrix molecule expressed in the mesenchyme around embryonic and adult epithelium, and in some epithelial tumours. Tenascin is a glycoprotein consisting of six identical disulphide-linked subunits of 190–230 kDa, but within each subunit there are structurally distinct regions. The part of the subunit which appears to be important for cell attachment resides on the C terminal side. In this region are a group of up to 11 fibronectin type III repeats containing a cell binding domain within the 10th and 11th repeat. On the N terminal side are 13 repeats with an EGF like motif, and isolated fragments from this region display anti-adhesive activity. The presence of two apparently opposing properties on the one molecule suggests that the interaction of cells with this molecule may depend upon which of the functional sites they are able to respond to.

The fate of cells is clearly determined by interactions with more than one class of matrix macro-molecule, but what combination of factors are required and how the information is specified remains to be determined. One way of approaching this problem has been through the use of *homotypic and heterotypic tissue grafting*; some remarkable examples of which were presented by Gerry Cunha (San Francisco). Heterotypic grafts of adult bladder epithelium and fetal urogenital sinus mesenchyme (UGM) result in the organotypic development of prostate epithelium. These grafts secrete prostatic specific antigens, enzymes, and initiate DNA synthesis in response to androgens. More significantly, certain types of mesenchyme were shown to influence the development of the R3327 androgen dependent Dunning prostatic carcinoma cell line. Notably the grafted mesenchyme from the urogenital sinus, seminal vesicles and uterus were able to induce this poorly differentiated tumour to develop well defined ductular structures that were filled with fluid secretion. These findings suggest that the properties of carcinoma cells may also be subject to controlling influences from the mesenchyme.

The importance of *epithelial-mesenchymal interactions* to the intestinal epithelium was also discussed by Patricia Simon-Assmann (Strasbourg). In this tissue these interactions are obligatory to induce differentiation of the epithelium and synthesis of a basal lamina. Using heterospecific tissue grafts and specific antiserum, it has been shown that the epithelium contributes heparan sulphate proteoglycans to the basal lamina, and the mesenchyme contributes collagen type IV, laminin and glycosaminoglycans. Differentiation of the epithelium *in vitro* has only been obtained in co-cultures (with direct contact to fibroblasts) in the presence of glucocorticoids. Studies with the colonic adenocarcinoma cell lines HT29 and CACO-2 have further shown that fibroblasts or defined matrix components can influence the cell–cell interaction and matrix depositions by intestinal tumour cells.

Cultured epithelial cells are generally poor models to study differentiation, due to a loss of function accompanying growth on plastic. In recent studies specific *gene induction* has been demonstrated in cultured epithelium and Mina Bissell (Berkeley) described how this had been achieved with the use of extracellular matrix and growth factors. Mammary epithelial cells (of the mid-pregnant mouse) synthesise milk proteins (e.g. casein, transferrin, whey acid protein and alpha lactalbumin) only when cultured in matrigel or isolated components of this matrix (e.g. laminin, collagen type IV and heparan sulphate proteoglycan). The matrix regulates these proteins at the level of gene expression and the synthesis, transport and degradation of these products. Cell substratum interactions also lead to reorganisation of the extracellular matrix itself and hormones such as TGF$\beta$ are potent inhibitors of milk protein synthesis. These complex interactions suggest a dynamic reciprocity in which the cell and the matrix acts as a single unit. This idea is supported by the presence of cell surface receptors (such as the integrins) which interact with extracellular matrix molecules, and via the cytoskeleton and nuclear matrix may ultimately influence mRNA and protein expression.

At the level of cell–cell interactions there are many processes which can influence the fate of cells, e.g. the formation of specific cell contacts, or interactions with the extracellular matrix and growth factors. Evidence to support this view was given by Terry Allen (Manchester) in time lapse video studies of 'long term' bone marrow, which allows the maintenance of *haemopoiesis in vitro*. These cultures contain many cell types whose interactions can be followed, such as erythroblasts which attach to the surface of macrophages, divide, and then undergo a synchronous process of enucleation to form erythrocytes. Large blanket cells in these cultures often cover many of the macrophages, and provide a suitable niche for granulopoiesis on their ventral surface. Once formed the mature granulocytes migrate up through the cell layer in a manner very similar to their egress from the bone marrow *in vivo*.

*Tractional forces* are essential to morphogenesis in the embryo, as in the condensation of mesenchyme in developing cartilage, somites, dermis and nephrons. Jonathan Bard (Edinburgh) explained the importance of these forces, and the role of cell–celll adhesion contacts in morphogenesis. In some embryonic tissues CAMS are expressed in a clear 'pre-pattern' and tractional forces play a subsidiary role in the morphogenesis of the tissue. However, in the formation of kidney nephrons tractional forces do seem to be essential. When embryonic kidney remnants are grown under conditions where cells are unable to exert traction, the mesenchyme fails to form aggregates that differentiate into nephrons. As traction is a random process, these observations suggest that pattern formation may be an intrinsic property of a developing aggregate and not necessarily dependent upon a 'pre-pattern'.

In the early stages of amphibian embryogenesis there are *inductive processes* which lead to major switches in cell differentiation. John Gurdon (Cambridge) showed how accessible these processes are to study, unlike similar events in early vertebrate development. Muscle development in the amphibian is a very early event and dependent upon a signal originated in the embryonic vegetal pole. The differentiation of muscle cells takes about nine hours and members of the actin gene family are expressed early in this process. Part of the actin gene consist of sequences responsive to inductive signals of muscle development, e.g. basic FGF and TGF$\beta$ are permissive factors for actin gene expression. A region of the actin gene promoter which is essential to obtain muscle development has homology to sequences which bind the Fos protein (and serum responsive elements). Although the proteins that bind this region are unidentified, evidence suggests there are several which may compete to achieve regulation of this gene during changes in differentiation and proliferation of the embryonic cells.

Martin Hooper (Edinburgh) described how pre-implantation *embryonal stem (ES) cells* can be used as a powerful model of cellular interactions in development. These cells are undifferentiated in culture (unless induced), and appear totipotent when placed into normal pre-implantation embryos, since they contribute cells to somatic tissues and occasionally the germ line. In communities of differentiating embryonic cells, inductive signals such as ions and small molecules may depend upon the expression of intercellular gap junctions. By combining normal ES cells, and variants deficient in cell communication, the importance of gap junctions in differentiation can be studied in culture. The incorporation of male ES cells into female embryos was also being used to study sex determination since the contribution of male cells (detected by a Y chromosome probe) appeared to play a role in the sexual development of the accessory sexual organs. The introduction of genes into the germ line by homologous recombination in ES cells, also raises the possibility of powerful new models to study familial cancers.

Recent developments showing the importance of cellular interactions in the development and function of the *thymus* were described by John Owen (Birmingham). Outside of the thymus, the proliferation of T cell precursors can be stimulated by IL-2 and IL-4, but these cells show no T cell receptor gene rearrangements. Signals which can lead to T cell receptor gene rearrangements seem to require the CD3 receptor, this receptor may also mediate signals for the negative selection of autoreactive T cells. Antibodies raised against this receptor complex can activate deletion of immature thymus cells by apoptosis, though normally this may be a function of the dendritic cell. In contrast, the thymic epithelial cells appear to be involved in the positive selection of certain T cells, particularly those expressing receptors to self MHC antigens. The extensive range of antigens expressed by the thymic epithelial cells is compatible with this process, although the selection takes place in an apparent (measurable) absence of these self-antigens.

In tissues many specialised cell types interact, but these different cell types also contain heterogeneous sub-populations which may influence the growth, differentiation and migration properties of other cells. Seth Schor (Manchester) presented a good example of this, describing the properties of *fetal-like fibroblasts* found in some patients with a predisposition to breast cancer. Unlike fibroblasts in normal adults, these cells are fetal-like because they migrate into three-dimensional collagen gels *in vitro*. The persistence of these cells into adulthood in some individuals could be a predisposing factor to the development of breast cancer. A 70 kDa migration stimulating factor (MSF) produced by fetal and fetal-like fibroblasts can induce adult fibroblasts to a migratory phenotype, and appears to act through stimulation of hyaluronic acid production. The inappropriate expression of hyaluronic acid may well contribute to pathological changes, since this molecule has been implicated in many processes such as angiogenesis, cell differentiation, proliferation and migration. The presence of detectable MSF in the serum of cancer patient indicates that this may also be a useful prognostic indicator.

Sampath Narayanan (Seattle) discussed the evidence and importance of heterogeneity among populations of *gingival fibroblasts*. On the basis of Clq binding, two populations of cultured fibroblasts can be distinguished. Up to 5% of the cells have a high affinity for Clq binding, and synthesise proteins and collagens at a much higher rate and are able to activate complement. In the presence of fresh serum or when fibroblasts are isolated from inflamed tissue the proportion of these cells can increase to more than 25%. Another population of fibroblasts shows an altered serum requirement, growing up to and beyond 40 passages (in heated plasma derived serum) partly due to an increased expression of growth factors. Clonal selection of these sub-populations was considered to be an important process during wound healing and could be an element in the progression of a tissue to neoplasia.

Both *microvasculature endothelium and pericytes* are required for angiogenesis, though little is understood about the nature of their interactions. Anna Schor (Manchester) described how both cell types retain some morphological similarities of their *in vivo* counterparts, when they are grown two-dimensionally on plastic surfaces *in vitro*. They also express different collagen types consistent with their function and position within a blood vessel, i.e. pericytes synthesise interstitial collagens type I and III (components of the outer basement membrane of blood vessels). However, in three-dimensional collagen gels *in vitro* the differences are not so well maintained. Single endothelial cells show a 'sprouting' morphology and spontaneously reassemble to form tubules analogous to blood vessels. The pericytes also form tubules when plated as aggregates, but both cell types now make large amounts of collagen IV and smaller amounts of collagens I and III and fibronectin. The phenotype of these two cell types can consequently be modified depending upon the nature of the cell–cell and cell matrix interactions.

The identification of multi-functional growth factors which act as signals for proliferation and differentiation has generated much interest, and although the potency of these factors is without question, how these messages are delivered and regulated is less clear. William Richardson (London) discussed the effects of *platelet derived growth factor (PDGF)* on the bipotential glial progenitor (0-2A) cells of the developing rat optic nerve. The proliferation of 0-2A cells is driven by homodimers of the PDGF-AA class, and these cells then give rise to oligodendrocytes and type 2 astrocytes in the first and second weeks of postnatal development respectively. The 02-A cells also bear the appropriate sub-class type A receptor for this growth factor, and only proliferate *in vivo* and *in vitro* for a limited period of time. After this the cells differentiate into oligodendrocytes, and loose responsiveness to PDGF. The exact mechanism by which the responsiveness is lost is not clear, but it is not due to a defect in the receptor or ability to activate cytosolic calcium release.

An informative review of the *transforming growth factor (TGF)-beta superfamily* was given by Lalage Wakefield (Bethesda). This family of factors is active in embryonic development, tissue repair, immunomodulation, inhibition of proliferation (particularly in epithelial cells) and induction of cell differentiation. Regulation of this potent factor usually occurs through its secretion in a latent precursor form, but the smaller (25 kDa) active form can be inactivated through reversible but rapid associations with beta-2-macroglobulin. The ubiquitous expression and growth inhibitory activity of TGF$\beta$ in epithelial cells of adult tissues, suggest that loss of response to this molecule could be an important step in cell transformation. However, many carcinoma cell lines remain sensitive to TGF$\beta$ *in vitro*, and their expression of TGF$\beta$ can be modulated, e.g. antioestrogens like tamoxifen inhibit the growth of the MCF-7 breast carcinoma line by stimulating the release of active TGF$\beta$. The growth inhibitory effects of tamoxifen in breast cancers may also be due to non-oestrogen receptor mediated events. Expression of active TGF$\beta_1$ has been observed in oestrogen receptor negative fibroblast cell lines treated with tamoxifen. In these cells regulation of the TGF$\beta$ message was at the post transcriptional level, suggesting a novel non-oestrogen receptor mediated pathway for the anti-oestrogens.

The *fibroblast growth factor (FGFs)* family have many functions and appear to act at local levels of cellular interactions; some FGFs bind to heparin (and heparin like matrix components) and are expressed locally in embryonic development. There are different forms of the FGFs, and Gordon Peters (London) summarised recent progress in characterising one of these, the product of the Int-2 oncogene. Apart from virally induced expression in the mouse mammary tumour, high levels of expression are also seen in some embryonic mesenchymal cells and in differentiating F9 teratocarcinoma cells. The Int-2 gene can be expressed in multiple transcripts and these have several potential promoter sequences, suggesting a complex regulation. However, these messages all encode the same translation product, which has a potential signal peptide sequence on the amino terminus (unlike other FGFs). Paradoxically the Int-2 product does not appear to be secreted, but is weakly mitogenic *in vitro* to 3T3 fibroblasts and mammary epithelial cells. These factors indicate that the Int-2 product may be restricted in its distribution, perhaps to presentation on the cell surface.

A talk which combined many aspects of the symposium was presented by Jean Paul-Thiery (Paris), who described the importance of cellular interactions in morphogenesis, *cancer invasion and metastasis*. Central to these processes are specific adhesion molecules (in epithelium desmosomes, cadhedrins, and CAM molecules) and matrix receptors (the integrin family). In embryonic development, mesencymal cells derived from the neural epithelium become migratory as they progressively loose cell–cell adhesion molecules, redistribute cell substratum receptors, and attach to different binding domains of fibronectin. The importance of fibronectin is demonstrated by the ability of GRGDS peptides to block these changes. Matrix components can also effect the migration of malignant cells; one rat bladder carcinoma line will convert to a migratory phenotype when plated on to different types of collagen, but not fibronectin or laminin. A reversible transition to this state is also induced by acidic FGF, but in combination acidic FGF and collagens induce the carcinoma cells to migrate and invade the gel. This suggests that cell–cell and cell matrix interactions are intimately linked and central to understanding normal cell development and progression to malignant and invasive states.

During the conference a variety of perspectives on cell interactions had been presented, and in the concluding discussion it was generally agreed that we need to move away from complex models and to ask more specific questions; but how should this be achieved? If a reductionist approach was adopted then how can the activity of a single type of matrix molecule be demonstrated using the present models systems? So far the constitution and structure of the extracellular matrix is still poorly understood and what is required is a better appreciation of how the chemical and physical aspects of the matrix influence cell shape and ultimately gene expression.

Central to this discussion was how the term differentiation is defined. In summarising the meeting, Michael Dexter (Manchester) outlined some aspects of this problem. The differentiation of a cell may be a 'stochastic' or deterministic process, but if it is stochastic then the effects of matrix can only be permissive, that is allowing a pre-ordained set of instructions, and if it is a deterministic process then the matrix may have more directive (instructive influences). In most of the model systems outlined during this symposium, the cells were already committed and many of the changes seen represent phenotypic modulation rather than differentiation. However, it may not be necessary to understand fully how these inductive signals (like growth factors) work, before applying them as agents to control the growth of normal or malignant cells.

G.S. Evans